# Real-world effectiveness of natalizumab treatment in patients with relapsing multiple sclerosis in Argentina and Chile

**DOI:** 10.1590/0004-282X-ANP-2020-0303

**Published:** 2021-05-01

**Authors:** Maria Celica Ysrraelit, Alejandro Caride, Vladimiro Sinay, Mario Rivera Kindel, Mario Javier Halfon, Liliana Patrucco, Raul Piedrabuena, Vanina Eleonor Diaz Aragunde

**Affiliations:** 1 FLENI Institute for Neurological Research Dr Raul Carrea Neurology Department Buenos Aires Argentina Institute for Neurological Research Dr Raul Carrea, FLENI, Neurology Department, Buenos Aires, Argentina.; 2 Hospital Aleman Department of Neuroscience Neuroimmunology Unit Buenos Aires Argentina Hospital Aleman, Department of Neuroscience, Neuroimmunology Unit, Buenos Aires, Argentina.; 3 Fundación Favaloro Hospital Buenos Aires Argentina Fundación Favaloro Hospital, Buenos Aires, Argentina.; 4 Clínica Dávila Región Metropolitana Chile Clínica Dávila, Recoleta, Región Metropolitana, Chile.; 5 British Hospital of Buenos Aires Buenos Aires Argentina British Hospital of Buenos Aires, Buenos Aires, Argentina.; 6 Hospital Italiano de Buenos Aires Buenos Aires Argentina Hospital Italiano de Buenos Aires, Buenos Aires, Argentina.; 7 Clínica Universitaria Reina Fabiola Servicio de Neurología Córdoba Argentina Clínica Universitaria Reina Fabiola, Servicio de Neurología, Córdoba, Argentina.; 8 Biogen S.R.L. Buenos Aires Argentina Biogen S.R.L., Buenos Aires, Argentina.

**Keywords:** Observational Study, Effectiveness, Multiple Sclerosis, Natalizumab, Latin America, Estudio Observacional, Efectividad, Esclerosis Múltiple, Natalizumab, América

## Abstract

**Background:**

The real-world effectiveness of natalizumab in people with relapsing multiple sclerosis (PwRMS) in Argentina and Chile has not been reported.

**Objective:**

To evaluate the effectiveness of natalizumab treatment in PwRMS in Argentina and Chile, in clinical practice.

**Methods:**

We conducted a multicenter retrospective and observational study. We reviewed the medical records of PwRMS who had been treated with natalizumab for at least one year, without any interruption in MS treatment that lasted more than 12 weeks. We analyzed changes in annualized relapse rate (ARR), Expanded Disability Status Scale (EDSS) score and magnetic resonance imaging (MRI).

**Results:**

We enrolled 117 PwRMS treated with natalizumab. Natalizumab treatment was associated with a significant reduction in ARR from baseline after one year and two years of treatment (from 1.97 to 0.06 and 0.09 respectively; p<0.01 at each time point). From baseline, EDSS scores were reduced by 0.71 and 0.73 points at one and two years, respectively (p<0.01). No worsening of disability was observed in 82.9 and 67.5% of PwRMS at one and two years, respectively. The improvement in disability was 44.4% at one year and 39.3% at two years. During natalizumab treatment, the number of relapse-related hospitalizations was significantly reduced (p<0.01). MRI lesions (new/enlarging T2 or gadolinium-enhancing) were significantly reduced, compared with baseline. No evidence of disease activity was observed in 65% at two years of natalizumab treatment.

**Conclusions:**

Natalizumab significantly reduced disease activity in PwRMS in Argentina and Chile, in clinical practice. Natalizumab also decreased the number of hospitalizations compared with pre-natalizumab treatment.

## RESUMEN

**Antecedentes:**

La efectividad de Natalizumab en personas con esclerosis múltiple recurrente (PwRMS) en Argentina y Chile no se ha reportado.

**Objetivo:**

Evaluar la efectividad del tratamiento con Natalizumab en PwRMS en Argentina y Chile en la práctica clínica.

**Métodos:**

Estudio multicéntrico, retrospectivo y observacional. Revisamos los registros médicos de PwRMS que fueron tratados con Natalizumab al menos 1 año, sin interrupción de tratamiento para EM durante más de 12 semanas. Analizamos los cambios en la tasa anualizada de recaídas (ARR), escala de discapacidad expandida (EDSS) y resonancia magnética (MRI).

**Resultados:**

Se incluyeron 117 PwRMS. El tratamiento con Natalizumab se asoció con una reducción significativa de la tasa anualizada de recaídas (ARR) cada 1 y 2 años (de 1.97 a 0.06 y 0.09, respectivamente; p<0.01 en ambos casos). El EDSS se redujo 0,71 y 0,73 puntos al año 1 y 2, respectivamente (p<0,01). No se observó empeoramiento del EDSS en 82,9 y 67,5% de los PwRMS al año 1 y 2, respectivamente. La mejoría del EDSS fue 44,4 y 39,3% al año 1 y 2, respectivamente. El número de hospitalizaciones se redujo significativamente (p<0,01). Las lesiones en MRI (nuevas/agrandadas en T2 o con realce con gadolinio) se redujeron significativamente en comparación con el valor basal. No se observó evidencia de actividad de la enfermedad en el 65% de los PwRMS a 2 los años.

**Conclusiones:**

Natalizumab redujo significativamente la actividad de la enfermedad en PwRMS de Argentina y Chile en la práctica clínica. Además, disminuyó el número de hospitalizaciones comparado con el tratamiento previo.

## INTRODUCTION

Multiple sclerosis (MS) is an inflammatory and neurodegenerative disease of the central nervous system (CNS) with estimated prevalences of 38.2 and 5.7 per 100,000 inhabitants in the city of Buenos Aires, Argentina, and in Chile, respectively^1^^,^^2^^,^^3^. MS is the leading cause of non-traumatic neurological disability in young adults worldwide^1^.

Natalizumab (Tysabri®, Biogen) is a humanized monoclonal antibody (an α4 integrin antagonist) that is indicated for treating people with relapsing multiple sclerosis (PwRMS) who have a poor response to first-line disease- modifying therapies (DMTs), and is indicated for patients who are treatment-naïve and present aggressive MS^4^^,^^5^.

The pivotal randomized controlled studies AFFIRM and SENTINEL provided evidence of the efficacy of natalizumab for its use in PwRMS^4^^,^^5^. In the phase III AFFIRM study^4^, natalizumab reduced the sustained progression of disability by 42% at two years and the relapse rate by 68% at one year. In the SENTINEL study^5^, the combination of natalizumab and interferon β1a reduced the sustained progression of disability by 24% at two years and the relapse rate by 54% at one year, in comparison with interferon β1a.

Several large observational studies in many countries of the European Union and also in the United States (US) have corroborated the effectiveness of natalizumab in real-world clinical practice^6^^,^^7^^,^^8^. Natalizumab has been commercially available in Argentina and Chile since 2010 and 2011, respectively. However, the effectiveness of natalizumab in clinical practice has not previously been studied in Argentina and Chile. Given the few data in our region, we aimed to evaluate the effectiveness of natalizumab in clinical practice in Argentina and Chile through collection of data on demographics, frequency of relapse-related hospitalizations, clinical observations and magnetic resonance imaging (MRI) from clinical records of PwRMS treated with natalizumab.

## METHODS

We conducted a multicenter, observational and retrospective study on data at 18 clinics in Argentina and Chile between May 2017 and February 2018. We reviewed all medical record databases of PwRMS, in accordance with the validated 2010 McDonald criteria^1^, on patients who were treated with natalizumab for at least one year, without any interruption in MS treatment that lasted more than 12 weeks. No data about adherence was obtained.

This study was approved by the local ethics committee of each participating center and oral or written informed consent was obtained from all participants. The study was conducted in accordance with the Declaration of Helsinki and Good Clinical Practice guidelines.

### Assessment and endpoints

For each patient, an ad hoc questionnaire was filled out in a single database that encompassed all the data from all the participating centers in Argentina and Chile and which included demographic, clinical and MRI data, as well as the information concerning natalizumab therapy.

Data collection included demographics (age and gender); clinical background and DMT use; incidence of relapses and relapse-related hospitalizations (before and after treatment with natalizumab compared with baseline ?one year prior to natalizumab use? and at one year and two years after the start of natalizumab use), disability measured by EDSS score^9^ (at baseline ?last EDSS score before the start of natalizumab use?, at one year ?12–15 months? and at two years ?24–27 months?); number of T1, T2 and gadolinium-enhancing (Gd+) lesions on MRI (at baseline ?last MRI before the start of natalizumab use?, between 6 and 12 months and between 18 and 24 months after the start of natalizumab use); and occurrence of a situation of no evidence of disease activity (NEDA; at 2 years after the start of natalizumab use).

The primary endpoint was whether there was a reduction in annualized relapse rate (ARR) during treatment with natalizumab (one year and two years) compared with the baseline (one year before the start of treatment with natalizumab), including the proportions of relapse-free patients at one year and two years. The secondary endpoints included measurements of MS disease activity such as MRI parameters and changes in EDSS score, number of relapse-related hospitalizations and overall NEDA^10^.

Relapses were defined as patient-reported or objectively observed events typical of a current or historical acute inflammatory demyelinating event in the CNS, with a duration of at least 24 hours, in the absence of fever or infection^1^. New or recurrent neurological symptoms which occurred within 30 days of the onset of another relapse were considered to be part of the same relapse^1^. Worsening of disability was defined as any increase in EDSS score from baseline and improvement of disability was defined as any decrease in EDSS score from baseline. Overall NEDA was analyzed post hoc and was defined as follows^10^: no increase in EDSS score, no relapses, no new Gd+ and/or new/enlarging T2 lesions between baseline and two years. EDSS and MRI scans were evaluated by neurologists with expertise in MS care. To reduce the risk of entry error with EDSS score reporting, the electronic case report form calculated an EDSS score based on the Kurtzke FS^9^ and ambulation scores that were entered. Because this was a retrospective multicenter study, MRIs were performed using different machines.

### Statistical analyses

Continuous data were expressed as means and standard deviation (±SD), median, maximum and minimum values. The categorical data were expressed as proportions. The Kolmogorov-Smirnov test was used to evaluate the normal distribution of variables (p<0.001). Changes in the ARR and MRI were analyzed using the Wilcoxon matched-pair signed-rank test and McNemar test on paired proportions, as appropriate. Poisson distribution and negative bimodal regression models were used to determine whether there was an association between predictor variables and the incidence rate ratio (IRR) of relapses at two years. Based on the data of the TOP study^8^, a reduction of 80% in ARR would be expected to be observed at two years after the start of natalizumab use. Assuming a data loss rate of 20%, at least 100 patients would be needed to provide 95% power to detect an 80% decrease in ARR, based on a simulated result (determination of sample size). Statistical analyses were performed using the STATA 12.0 software (StataCorp TX, US). For all the analyses, the significance level was established as p<0.05.

## RESULTS

**Figure 1:**
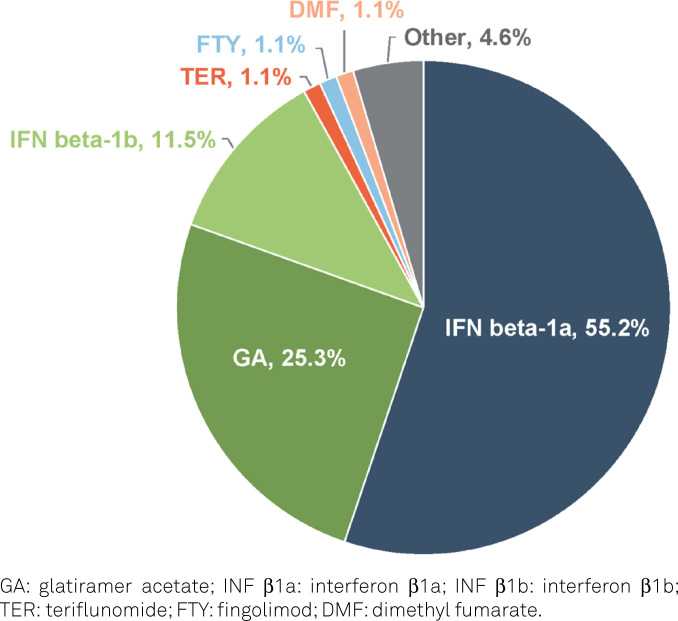
Initial disease-modifying therapy used by patients who reported prior disease-modifying therapy use (n=87).

We enrolled 117 PwRMS who were being treated with natalizumab. At baseline, 59.8% were female with a mean age of 35.4 years (±10.4) and a median MS duration before starting natalizumab of 36 months (IQR: 11–84). Prior DMT use was reported by 75.1% of the PwRMS. As shown in Figure 1, most patients had started their MS treatment with injectable interferons (IFN) (58 out of 87 patients) or glatiramer acetate (22 out of 87 patients). The demographic data and clinical features at baseline are summarized in Table 1.

**Table 1:** Baseline clinical characteristics of the study population.

Characteristic		Patients with characteristic (n=117)
Female, n (%)		70 (59.8)
Age mean (±SD), years		35.4 (±10.4)
Disease duration prior to diagnosis, median (range), months		36 (11-84)
DMT use prior to natalizumab, n (%)		87 (75)^a^
Immunosuppressant use prior to natalizumab, n (%)		7 (8.1)^b^
EDSS, mean (±SD)		3.77 (±1.84)
Relapses in year prior to natalizumab, mean (±SD)		2.0 (±1.2)
Relapse-related hospitalizations in previous year, mean (±SD)		1.54 (±0.85)^c^
Patients with MRI lesions, n (%)^d^	
	T1 lesions T2 lesions Gd+ lesions	94 (83.2) 114 (99.1) 73 (63.5)
MRI lesions per patients, mean (±SD)^e^		
	T1 lesions T2 lesions Gd+ lesions	6.8 (±3.3) 18.5 (±8.7) 2.7 (±3.3)

SD: standard deviations, EDSS: Expanded Disability Status Scale, DMT: disease-modifying therapy, MRI: magnetic resonance imaging, Gd+: gadolinium-enhancing.

a n=116;

b n=87;

c 83 patients were hospitalized due to relapse;

d data on T1, T2 and Gd+ lesions were available for 113, 115 and 115 patients, respectively;

e data on T1, T2 and Gd+ lesions available for 94, 109 and 115 patients, respectively.

### Endpoints

#### Relapses and hospitalizations

**Figure 2:**
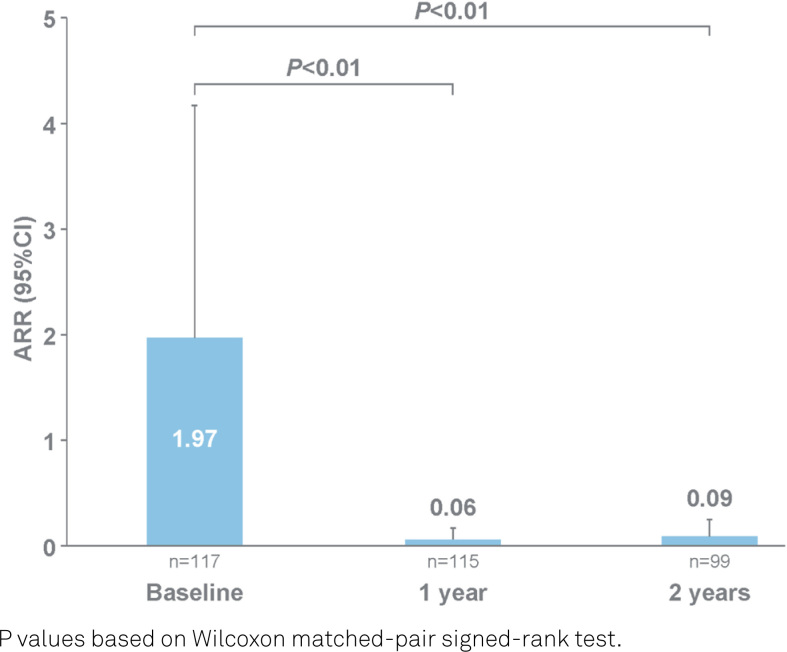
Mean annualized relapse rate in study population at baseline, one year and two years.

At baseline, the patients had a mean ARR of 1.97 (95% confidence interval [95%CI] 1.75–2.20). As shown in Figure 2, natalizumab treatment was associated with a significant reduction in ARR from baseline after one year and two years of treatment (from 1.97 to 0.06 and 0.09 respectively; p<0.01 at each time point).

The proportion of patients free from relapses was significantly higher after one year (93.9%; 95%CI, 87.9–97.5; p<0.01) and two years (92.9%; 95%CI, 86.0–97.1; p<0.01) of natalizumab treatment, in comparison with the year before starting natalizumab treatment (7.7%; 95%CI, 3.6–14.1). In the year before starting natalizumab use, 83 out of 117 patients had experienced relapse-related hospitalizations (Table 1). Natalizumab treatment reduced the number of relapse-related hospitalizations to one occurrence at one year (n=114) and three occurrences at two years (n=98), which was a significant (p<0.01) reduction in each case. As shown in Table 2, no association between predictor variables and the IRR of relapses at two years was observed after applying negative binomial regression analysis.

**Table 2:** Patients who became free from clinical and radiological disease activity over two years of natalizumab treatment.

Assessment*		Patients without disease activity at 2 years n/N (%)
Clinical assessment		
	No increase in EDSS score Free from relapse over 2 years	77/95 (81.1) 87/99 (87.9)
MRI assessment		
	No new Gd+ lesions over 2 years No new/newly enlarging T2 lesions	90/98 (91.8) 79/90 (87.8)
Overall NEDA		57/87 (65.5)

EDSS: Expanded Disability Status Scale, MRI: magnetic resonance imaging, Gd+: gadolinium-enhancing, NEDA: no evidence of disease activity.

* All assessments were for the interval between baseline and two years. Proportions represent number of patients meeting specified criteria divided by total number of patients with data available at baseline, one year and two years. Patients with overall NEDA were defined as those free from disease activity, as assessed through the four clinical and radiological criteria above.

### Disability

**Figure 3:**
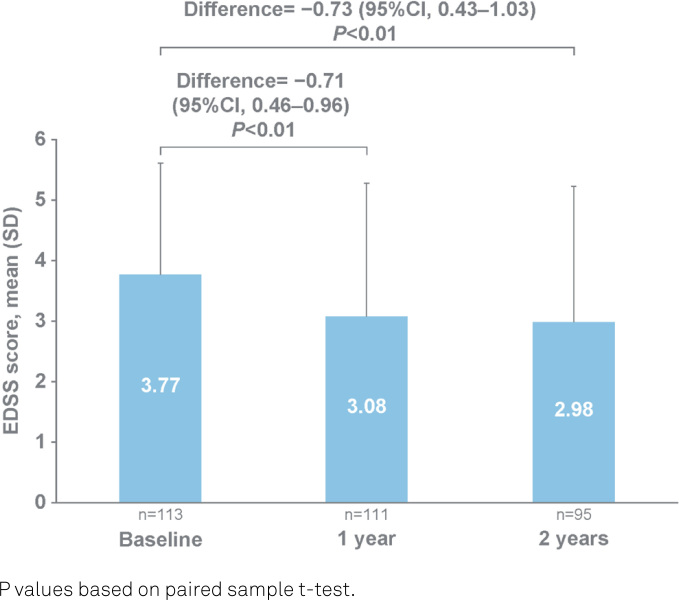
Mean Expanded Disability Status Scale scores at baseline, one year and two years.

From baseline, the mean EDSS scores were reduced by 0.71 points (95%CI 0.46–0.96) at one year and 0.73 points (95%CI 0.43–1.03) at two years, as illustrated in Figure 3.

**Figure 4:**
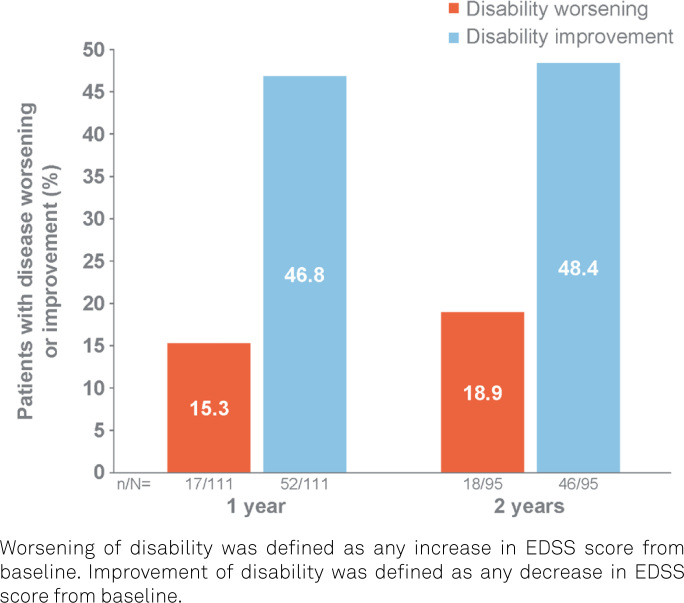
Proportions of patients with worsening and improvement of disability at one year and two years.

The proportion of the PwRMS with worsening of disability was 15.3% at one year and 18.9% at two years. The proportion of the PwRMS with improvement of disability was 46.8% at one year and 48.4% at two years, as illustrated in Figure 4.

### Magnetic resonance imaging

**Figure 5:**
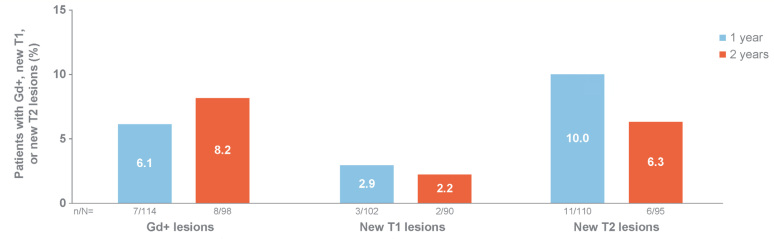
Patients with MRI activity after one year and two years of natalizumab treatment.

At baseline, 94 out of 113 (83.2%), 114 out of 115 (99.1%) and 73 out of 115 (63.5%) of the PwRMS had T1, T2 and Gd+ lesions, respectively (Table 1). As shown in Figure 5, at one year and two years the proportion of the PwRMS treated with natalizumab who still presented Gd+ lesions was significantly lower, compared with baseline (baseline: 73 out of 115 [63.5%]; one year: 7 out of 114 [6.1%]; and two years: 8 out of 98 [8.2%]; p<0.01 at each time point), as assessed by the McNemar test on paired proportions. After one year of natalizumab treatment, new T1 and T2 lesions were observed in 2.9 and 10.0% of the patients, respectively. After two years of treatment, 2.2 and 6.3% of the PwRMS had new T1 and T2 lesions, respectively.

### No evidence of disease activity

In total, 65.5% of the PwRMS met the criteria for overall NEDA between baseline and two years (Table 2). Between baseline and two years, 81.1% of the patients had no increase in EDSS score and 87.9% of the patients were relapse-free. Over the same span, 91.8% of the patients had no new Gd+ lesions and 87.8% had no new or newly enlarging T2 lesions.

## DISCUSSION

The findings observed in this study on real-world evidence, which was carried out in Argentina and Chile, were consistent with data from previous studies on natalizumab conducted in other regions. We observed that natalizumab was effective over a two-year period in terms of disease activity, measured by reduced relapse rate, stable EDSS and reductions in new or enlarging MRI lesions.

Over the two-year duration of the phase 3 AFFIRM study^4^ on PwRMS, natalizumab monotherapy demonstrated consistent efficacy in the overall study population and across multiple subgroups of patients that had been predefined on the basis of demographic and baseline disease characteristics, including age, sex, number of brain MRI lesions, disability status and number of relapses in the prior year^4^^,^^11^. Nonetheless, while the AFFIRM^4^ trial established the safety and efficacy of natalizumab, randomized controlled trial populations may not completely represent patients treated in a clinical practice setting^7^^,^^12^.

Therefore, data are needed to confirm the effectiveness of natalizumab in clinical practice, particularly in our region (Latin American populations), since previous data have come principally from Europe and North America^6^^,^^7^^,^^8^, and there were no data on any large multicenter cohort of Latin American patients, who would be expected to present differences in comparison with patients from these other regions. Many studies seeking real-world evidence have been and are being published, which are providing valuable information on natalizumab effectiveness in different clinical practice scenarios^7^. However, not all of them use the same outcome measurements.

In this regard, the Tysabri Observational Program (TOP) is an ongoing, open-label, multinational, multicenter, prospective, observational study conducted in Europe, Australia, Canada and Argentina^8^. Its results provide information on the long-term safety of natalizumab and its impact on ARR and EDSS progression in PwRMS after up to 10 years of treatment^13^. Unfortunately, it gives no data on MRI parameter outcomes. Unlike the AFFIRM population, in which the majority of the patients were treatment-naïve^4^, the majority of the patients in TOP (about 90%) and those in our study (75%) had been previously treated with a DMT and/or immunosuppressant therapy. Consistent with other studies from Europe and North America (ARR between 1.3 and 2.5)^14^^,^^15^^,^^16^^,^^17^^,^^18^^,^^19^^,^^20^^,^^21^^,^^22^^,^^23^^,^^24^^,^^25^^,^^26^^,^^27^^,^^28^^,^^29^^,^^30^^,^^31^, the mean number of relapses prior to natalizumab treatment initiation was similar between our patient population (2.0) and the TOP study (1.99)^8^, and considerably higher than the number among patients receiving natalizumab in the AFFIRM study (1.53)^4^. We found a similar situation in comparing the baseline EDSS scores between these populations: the mean baseline EDSS score among AFFIRM patients was 2.3^4^, while in TOP and our study population the scores were 3.5^8^ and 3.7, respectively. In addition, ranges from 3.7 to 4.8 were also reported in a review of observational studies^7^. These differences gain importance in everyday practice and should be taken into consideration, given that quite often patients in clinical settings do not match those studied in the pivotal trials.

In the present study, we observed that treatment with natalizumab significantly reduced the number of relapses at one and two years (93.9 and 92.9%, respectively). This was comparable with the 90.7% reduction in ARR achieved in TOP^32^ and 83.2% in a recently published study in the US^6^, as well as in other observational studies^14^^,^^15^^,^^16^^,^^17^^,^^18^^,^^19^^,^^20^^,^^21^^,^^22^^,^^23^^,^^24^^,^^25^^,^^26^^,^^27^^,^^28^^,^^29^^,^^30^^,^^31^. In addition, the TOP study reported that PwRMS treated with natalizumab presented associations with reductions in relapse-related hospitalizations and steroid treatment, despite longer follow-up in the on-natalizumab period than in the pre-natalizumab period^33^. In this regard, PwRMS treated with natalizumab in Argentina and Chile experienced significant reductions in relapse-related hospitalizations at one and two years. A real-life study on a Swiss cohort^20^ demonstrated that the effect of natalizumab on ARR reduction not only depended on disease activity at baseline, but also was effective in PwRMS after a long period of active disease (one and two years before baseline), thus suggesting that natalizumab presented sustained efficacy that was not derived from bias concerning the evaluation of baseline data.

Regarding disability, we observed that the decrease in EDSS score was greater than what was reported in Europe^21^^,^^26^^,^^30^, but lower than the results achieved in a study in Kuwait^34^. In addition, observational studies^7^^,^^26^^,^^27^^,^^28^^,^^29^^,^^30^^,^^31^^,^^32^^,^^33^^,^^34^^,^^35^ have reported that between 9 and 57% of PwRMS experienced improvement of disability, while 4‒17% had worsening of disability, which is consistent with our results. In this regard, a post hoc analysis within AFFIRM reported that PwRMS treated with natalizumab increased their cumulative likelihood of 12-week confirmed improvement of disability, compared with placebo at two years^36^.

When the MRI endpoints were analyzed, we found robust decreases in all parameters of disease activity. Similar to our results, the findings from other cohorts showed that the mean number of Gd+ lesions was reduced by 78‒93.1%^6^^,^^21^^,^^28^ after natalizumab treatment compared with pre-natalizumab, and no new or enlarging T2 lesions was reported in up to 95%^21^. Combination of these clinical and radiological outcomes resulted in a high proportion (65.5%) of patients reaching NEDA at two years. A recently published study (STRIVE study)^6^ reported that 44.4% of PwRMS who started natalizumab treatment early exhibited overall NEDA at two years. In addition, similar results (33‒63%)^14^^,^^15^^,^^16^^,^^17^^,^^18^^,^^21^^,^^28^^,^^35^ have been found in other real-world observational studies after mean follow-ups ranging from 1.3 to 2 years.

The present study had some limitations. It was a retrospective study with possible selection bias. Although careful data collection and patient follow-up were developed in each center, to decrease the possibility of potential information bias, there were some missing data. Additionally, when EDSS scores were collected, any change regardless of magnitude was counted as worsening or improvement and without confirmation; therefore, these results should be interpreted accordingly. In addition, we included relatively small numbers of patients in this analysis, with a lack of randomization and no internal comparator or control group. The patients included were from main MS centers of both countries, which may have generated an analysis bias. Lastly, although this was a cohort from two countries, it may not reflect the entire Latin American population.

Despite these limitations, the results from our study highlight the consistent effectiveness of natalizumab in clinical practice settings and provide local information in a cohort of Argentinean and Chilean patients. This study, along with TOP and other observational registries of natalizumab-treated patients^6^^,^^7^^,^^15^, will continue to generate valuable data on the long-term safety and effectiveness profile of natalizumab. These data will help in real-life decision-making when choosing the best treatment option for our patients.
